# Europe as a secondary distribution hub in the worldwide invasion of the potato cyst nematode *Globodera rostochiensis*

**DOI:** 10.1038/s41598-024-64617-0

**Published:** 2024-06-17

**Authors:** Magali Esquibet, James M. Mwangi, Sebastian Kiewnick, Xiaohong Wang, Benjamin Mimee, Nurul Dwi Handayani, Wim Bert, Johannes Helder, John Wainer, Itaru Sakata, Nathan Garcia, Eric Grenier, Josselin Montarry

**Affiliations:** 1 IGEPP, INRAE, Institut Agro, Univ Rennes, Le Rheu, France; 2 Department of Biological Sciences, Chuka University, Chuka, Kenya; 3 Julius Kühn-Institut, 38104 Braunschweig, Germany; 4 US Department of Agriculture, Agricultural Research Service, Robert W. Holley Center for Agriculture and Health and School of Integrative Plant Science, Cornell University, Ithaca, NY USA; 5 Agriculture and Agri-Food Canada, Saint-Jean-sur-Richelieu, QC Canada; 6 Nematology Research Unit, Department of Biology, Ghent University, Ghent, Belgium; 7 Laboratory of Nematology, Wageningen University, Wageningen, The Netherlands; 8 AgriBio Centre, Agriculture Victoria Research, Melbourne, Australia; 9 Hokkaido Agricultural Research Centre (HARC), National Agriculture and Food Research Organization (NARO), Hokkaido, Japan; 10 Nematology Unit, Plant Health Laboratory, ANSES, 35653 Le Rheu, France; 11 Indonesian Agricultural Quarantine Agency, Ministry of Agriculture, E Building 5th Floor, Jl. Harsono RM, 3 Ragunan, Jakarta, 12550 Indonesia

**Keywords:** Evolutionary biology, Population genetics, Molecular ecology

## Abstract

The potato cyst nematode *Globodera rostochiensis* originates from the Andean Mountain region in South America and has unintentionally been introduced to all inhabited continents. Several studies have examined the population genetic structure of this pest in various countries by using microsatellite markers. However, merging microsatellite data produced from different laboratories is challenging and can introduce uncertainty when interpreting the results. To overcome this challenge and to explore invasion routes of this pest, we have genotyped 22 *G. rostochiensis* populations from all continents. Within populations, the highest genetic diversity was observed in the South American populations, the European populations showed an intermediate level of genetic diversity and the remaining populations were the less diverse. This confirmed pre-existing knowledge such as a first introduction event from South America to Europe, but the less diverse populations could originate either from South America or from Europe. At the continental scale, STRUCTURE genetic clustering output indicated that North America and Asia have experienced at least two introduction events. Comparing different evolutionary scenarios, the Approximate Bayesian Computation analysis showed that Europe served as a secondary distribution centre for the invasion of *G. rostochiensis* into all other continents (North America, Africa, Asia and Oceania).

## Introduction

Nematoda constitutes a Metazoan phylum that includes free-living species, such as the model organism *Caenorhabditis elegans*, as well as many animal- and plant-parasitic species. Plant-parasitic nematodes cause considerable economic losses in agriculture. Every crop can be infested by at least one plant-parasitic nematode species, and the worldwide crop losses due to plant-parasitic nematodes have been estimated around $173 billion per year^1^. The most important plant-parasitic nematodes are root-knot nematodes (genus *Meloidogyne*) and cyst nematodes (genera *Heterodera* and *Globodera*)^2^. The latter are particularly difficult to eradicate because they form cysts that can persist many years in soils. Cyst nematodes are sedentary endoparasitic nematodes. They enter plant roots as second stage juveniles (J2) and establish a specialized feeding structure. Subsequently, adult males and females develop and the males leave the root to mate with females. The females continue to feed and when egg development is completed, they die and form a cyst containing hundreds of eggs, with one juvenile per egg. Being relatively small soil-borne parasites, the active dispersal ability of juveniles is limited to short distances^3^. However, their survival form, the cyst, can be dispersed over large spatial scale, either through natural means such as wind, water, and wildlife or through the transportation of soil and tubers due to human activities^4–6^.

Potato cyst nematodes (PCN) comprise two major species, *Globodera rostochiensis* and *G. pallida*, which are economically important pests of potatoes and are subject to strict regulations in many countries. *Globodera rostochiensis* was reported by EPPO (European and Mediterranean Plant Protection Organisation) as present in 76 countries around the world: 42 in Europe, 13 in Asia, 10 in America (4 in North America and 6 in South America), 8 in Africa and 3 in Oceania (EPPO Global Database, 08 September 2023). The Andean region is not only the centre of origin of potatoes but also the home to many potato pathogens, including plant-parasitic nematodes that attack this crop. *Globodera rostochiensis* is widely distributed in the Andean region: Peru, Ecuador, Colombia, Bolivia, Venezuela and Chile^7^. It was introduced around 1850 to Europe, probably through the importation of tubers from South America^8,9^. In North America, *G. rostochiensis* was first detected in New York state in 1941^10^ and in the western part of Canada in 1962^11^. The pest was detected more recently, in 2006, in Quebec, the eastern part of Canada^12^. In Japan, it was first identified in 1972^13^. This nematode was also reported in Lebanon^14^ and more recently, in several China provinces^15^. In Australia, *G. rostochiensis* was detected for the first time near Perth in 1986^16^, and this population was subsequently successfully eradicated^17^. However, it was detected in Victoria, near Melbourne (i.e., more than 3000 km from Perth) in 1991^18^. *Globodera rostochiensis* was also identified in Indonesia in 2003^19^. In Africa, *G. rostochiensis* was already known in the North (e.g., Algeria, Egypt and Tunisia) and in South-Africa^20^. In 2015, *G. rostochiensis* was first reported in East-Africa in Kenya^21^ and later in Rwanda and Uganda^22,23^.

Over the past 10 years, several population genetic studies were performed utilizing microsatellite markers (polymorphic neutral DNA markers that consists of short DNA repeat units) to explore genetic diversity and relationships among *G. rostochiensis* populations. Boucher et al.^24^ developed a set of 12 polymorphic microsatellite markers, and their results, based on the genotyping of 15 populations from South America, North America and Europe, showed that *G. rostochiensis* was introduced to North America at least twice. A few years later, three studies explored the origin of *G. rostochiensis* populations sampled in Kenya^25^, Australia^26^ and Indonesia^27^. Using six Kenyan populations and the same 12 microsatellite markers, Mwangi^25^ showed that the Kenyan populations formed a distinct genetic cluster separate from the populations studied by Boucher et al.^24^. Using nine of the 12 microsatellite markers, the results of Blacket et al.^26^ supported a probable single introduction and indicated that the Australian populations were genetically distinct from populations previously sampled worldwide. Ten markers of the 12 developed by Boucher et al.^24^ were found to be polymorphic in Indonesian *G. rostochiensis* populations (five populations from North Sumatra and two populations from East Java). The resulting genetic diversity and structure analyses suggested that the origin of North Sumatra cysts was East Java^27^, but the origin of the latter was not explored. Using whole genome resequencing data, a more recent study confirmed that *G. rostochiensis* was first introduced to Java and soon after dispersed from Java to Sumatra^28^.

Merging microsatellites data produced in different laboratories, i.e., using distinct fluorescent dyes, PCR conditions, *Taq* Polymerases, sequencers, and protocols to score alleles, could introduce uncertainty regarding the interpretation of the results (e.g.,^29–32^). When a newly sampled population was found genetically distant from previous ones, it can be challenging to distinguish the true genetic divergence from effects resulting from technical differences between laboratories. Moreover, the number of microsatellite markers used varied among the studies mentioned above.

To generate an accurate and extensive dataset on a global scale, *G. rostochiensis* populations were collected from different parts of the world and all populations were genotyped, from DNA extraction to allele-size reading, in a single laboratory. To minimize reading errors, allele sizes were identified using the automatic calling and binning procedure and completed by a manual examination by only one reader. This study aimed to explore the genetic features, genetic relationships, and the origin and routes of *G. rostochiensis* invasion worldwide using 22 populations from North and South America, Africa, Europe, Asia, and Oceania.

## Results

### Genetic features of *G. rostochiensis* populations

Using a set of 11 microsatellite markers, we identified 77 alleles among the 22 *G. rostochiensis* populations that were genotyped (i.e., among the 793 individuals without missing data), ranging from four (for Gp116, Gp118 and Gp135) to 16 (for Gr90) alleles per locus. Forty private alleles (alleles present in only one population) were detected, 37 being from South American populations.

The median number of individuals per population was 35 and the population having the lowest number of individuals was the Jap-Ho population (n = 29) (Table 1). The South American populations were the most diverse: the allelic richness (Ar), estimated on a reduced sample of 29 individuals, ranged from 1.72 to 3.97 alleles per locus, and the unbiased expected heterozygosity (H_nb_) ranged from 0.235 to 0.548 (Table 1). The highest genetic diversity was found in populations B2 and B4 from Bolivia. A low genetic diversity, with H_nb_ lower than 0.1, was observed within Kenyan and Australian populations, as well as within the two Indonesian populations from North Sumatra, the US population from New York State, the Japanese and one European population (CZ). The remaining European populations exhibited an intermediate genetic diversity: the allelic richness ranged from 1.54 to 1.70 alleles per locus and the unbiased expected heterozygosity from 0.149 to 0.155 (Table 1).

**Table 1:** Genetic diversity indices (H_nb_ and Ar) and deviation from random mating (*F*_IS_) for each *Globodera rostochiensis* population.

Country	Continent^1^	Code	n^2^	H_nb_	Ar (n = 29)	*F*_IS_^3^
Bolivia (Capaña)	S-A	B2	32	0.548	3.97	0.078
Bolivia (Tiraque)	S-A	B4	33	0.396	3.16	0.056
Peru (Huancane)	S-A	267	37	0.235	1.96	0.081
Chile (La Serena)	S-A	3346	40	0.250	1.72	0.002
Czech Republic (Svojše)	Europe	CZ	36	0.066	1.49	0.041
France (Dunkerque)	Europe	Dunk	46	0.155	1.61	0.032
Netherlands (Wageningen)	Europe	NL	35	0.151	1.54	0.002
Portugal (Montalegre)	Europe	Port	47	0.149	1.70	0.029
Canada, Quebec (St Amable)	N-A	Ama	38	0.147	1.43	0.043
United States (New York)	N-A	US	39	0.005	1.13	− 0.000
Kenya, (Haraka)	Africa	HAR2	31	0.097	1.62	0.277 *
Kenya, (Kinangop)	Africa	KIN2	31	0.098	1.54	0.070
Kenya, (Tigoni)	Africa	TGN	32	0.065	1.35	0.129
Kenya, (Rironi)	Africa	RIR	32	0.023	1.17	− 0.112
Indonesia, Sumatra (Lingga Julu)	Asia	NRK4	39	0.073	1.52	− 0.025
Indonesia, Sumatra (Suka Ndebi)	Asia	NRK6	38	0.078	1.36	− 0.046
Indonesia, Java (Sumber Brantas)	Asia	NRM1	39	0.100	1.41	− 0.046
Indonesia, Java (Krajan)	Asia	NRM2	33	0.161	1.72	− 0.046
Lebanon (Beyrouth)	Asia	Leb-Be	34	0.120	1.36	− 0.067
Japan, Hokkaido (Kutchan)	Asia	Jap-Ho	29	0.009	1.18	0.667 *
Australia, Victoria (Cora Lynn)	Oceania	CL	35	0.089	1.26	0.212
Australia, Victoria (Thorpdale)	Oceania	TH	37	0.052	1.34	0.051

^1^S-A for South-America and N-A for North-America.

^2^n is the number of genotyped individuals per population.

^3^Stars indicate that *F*_IS_ is significantly different from zero.

Regarding the deviation from the random mating hypothesis, 20 populations were at the Hardy–Weinberg equilibrium (i.e., *F*_IS_ not significantly different to zero). Only one Kenyan population, HAR2, and the Jap-Ho population showed strong heterozygote deficits, with significant positive values of *F*_IS_: 0.277 and 0.667, respectively (Table 1).

### Genetic differentiation between *G. rostochiensis* populations

**Figure 1:**
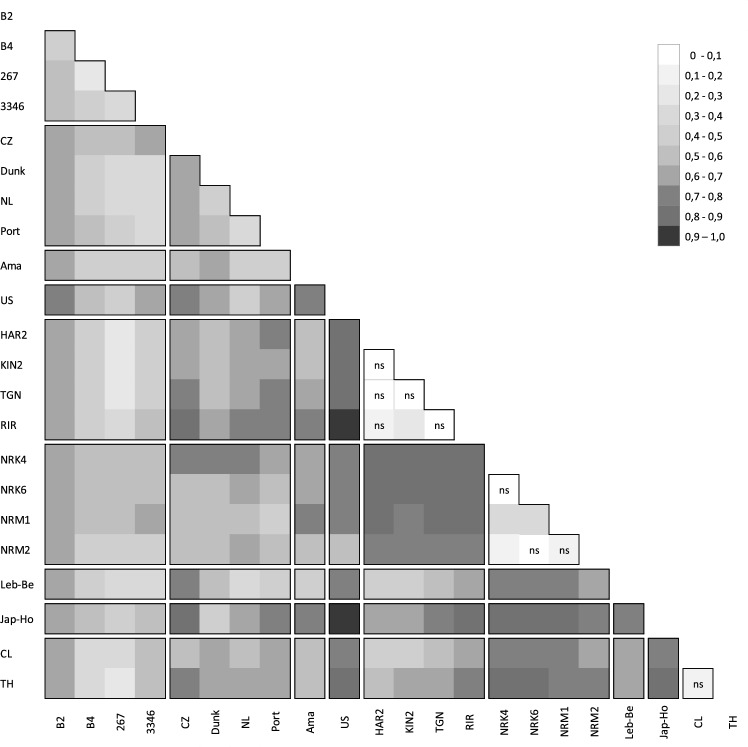
Matrix of pairwise *F*_ST_ between the 22 *Globodera rostochiensis* populations. Populations were separated according to the different groups constituted for the ABC analyses. ns indicates non-significant differentiation between two populations.

Among the 231 pairwise comparisons, the *F*_ST_ values, ranging from 0 to 0.91 (Fig. 1), were significant except for nine population pairs comparing populations sampled in the same country: Kenya, Indonesia or Australia. Overall, the genetic differentiation between the nine different defined groups, i.e., the two putative sources (South America and Europe) and the seven target populations or pools of populations (Ama, US, Kenya, Indonesia, Lebanon, Japan and Australia) was high and significant. Whereas for the *F*_ST_ calculated within each defined group, two cases were distinguishable: (i) the genetic differentiation was significant among populations from South America and from Europe, (ii) the genetic differentiation was low and mainly not significant among populations from Kenya, Indonesia and Australia. This result clearly indicated that the nematode dispersion within South America and the introduction of *G. rostochiensis* into Europe were much older events than the introduction to the other parts of the world.

### Genetic structure among *G. rostochiensis* populations

**Figure 2:**
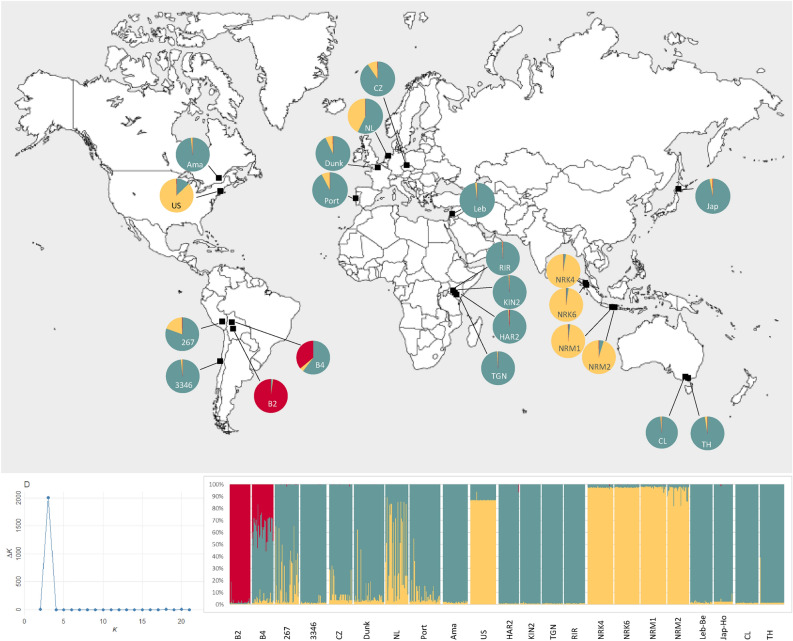
Bayesian clustering analysis (STRUCTURE) of the 793 *Globodera rostochiensis* individuals from the 22 populations collected in different continents. Assignment probabilities of individuals are presented for K = 3, i.e., the most likely number of clusters statistically determined using ΔK method (bottom-left graph). On the bottom-right graph, each vertical line represents an individual for which the genetic assignment is partitioned into three clusters, represented by three colors (cluster 1 in green, cluster 2 in yellow and cluster 3 in red), and vertical white lines separate each of the 22 populations. The map at the top shows the geographical location of the 22 *G. rostochiensis* populations and their membership proportion of clusters.

The Bayesian clustering analysis identified K = 3 as the optimal number of genetic clusters, with a high proportion of individuals well assigned to one cluster. Seventy-six percent of individuals were assigned to one cluster with a percentage of assignation higher than 95% (54% to cluster 1, 18% to cluster 2 and 4% to cluster 3). The four Kenyan, the two Australian, the Canadian, the Lebanese, the Japanese and the Chilean populations were entirely assigned to cluster 1 (Fig. 2). All the Indonesian populations and the US population were clearly assigned to cluster 2, even if the percentage of assignation was a little smaller for individuals of the US population (Fig. 2). The European populations (CZ, Dunk, NL and Port) and the Peruvian population were mainly assigned to cluster 1 but the number of individuals with a percentage of assignation higher than 95% was quite low (e.g., only 11% of the individuals from population NL). The remaining individuals were assigned to both cluster 1 and cluster 2 (Fig. 2). The two Bolivian populations had a distinct assignment from all other populations and corresponded alone to cluster 3. B2 was completely assigned to cluster 3 (31 out 32 individuals with a percentage of assignation higher than 95%), while all the individuals of B4 were assigned either to cluster 1 or 3 (Fig. 2).

### Inferences of invasion scenarios

**Figure 3:**
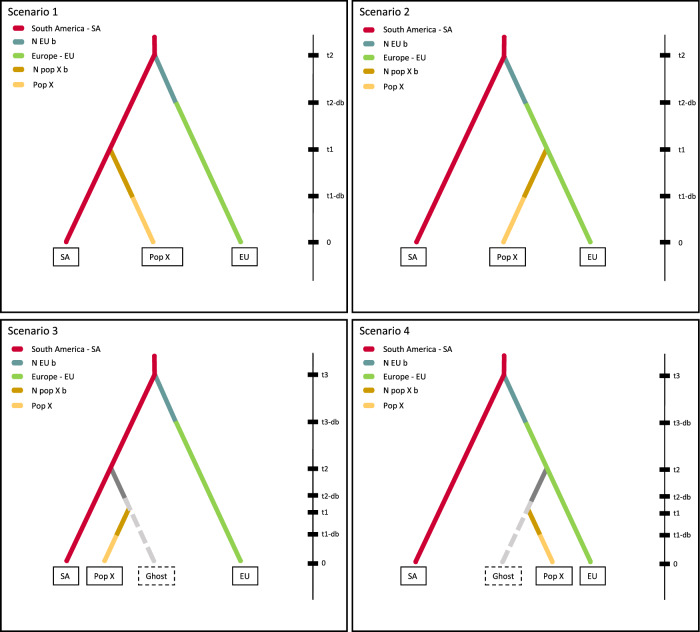
The four population divergence scenarios tested by the Approximate Bayesian Computation (ABC) approach using each target population (Canada, United States, Kenya, Indonesia, Lebanon, Japan and Australia). Since an invasive population generally starts with a few individuals, an initial size reduction (noted here ‘N pop X b’ for each target population and ‘N EU b’ for population Europe) was added. Times are not to scale, 0 indicates the present day and t on top of the bar indicates the oldest split, bd illustrates the duration of the bottleneck period. A ghost population is a possible unsampled source population.

The Approximate Bayesian Computation (ABC) was used here to compared four evolutionary scenarios that can explain observed data (Fig. 3). The main objective was to determine whether the origin of each target population is from South America or Europe. The DIYABC results indicated, with the posterior probability of scenario S2 higher than those for the other scenarios, that all the studied populations (Ama, US, Kenya, Indonesia, Leb-Be, Jap-Ho and Australia) were originated from Europe after the initial introduction into this continent (Table 2). The 95% confidence intervals (CIs) for scenario S2 did not overlap with those for the other scenarios (Table 2). These results also ruled out the possibility that a given population diverged from a ghost population which itself originated from Europe earlier in time.

**Table 2:** Results of the ABC analyses to infer the origin of each target genetic unit. Scenarios S1 and S3 tested a South American origin and scenarios S2 and S4 a European origin. The effect of a ghost population was tested by scenarios S3 and S4. The table shows the posterior probability of each scenario, with 95% confidence intervals in brackets, and bold numbers indicate the most highly supported scenario for each target unit.

Competing scenario	Origin	Canada (1 population)	USA (1 population)	Kenya (4 populations)	Indonesia (4 populations)
S1	South America	0.00 [0.00, 0.01]	0.01 [0.00, 0.01]	0.02 [0.00, 0.48]	0.00 [0.00, 0.01]
S2	Europe	**0.95 [0.94, 0.96]**	**0.95 [0.94, 0.96]**	**0.87 [0.82, 0.93]**	**0.92 [0.89, 0.94]**
S3	South America and unsampled source population	0.00 [0.00, 0.00]	0.00 [0.00, 0.00]	0.01 [0.00, 0.43]	0.00 [0.00, 0.00]
S4	Europe and unsampled source population	0.04 [0.03, 0.05]	0.04 [0.03, 0.05]	0.10 [0.00, 0.49]	0.08 [0.05, 0.10]

Competing scenario	Origin	Lebanon (1 population)	Japan (1 population)	Australia (2 populations)	
S1	South America	0.01 [0.01, 0.02]	0.01 [0.00, 0.01]	0.01 [0.00, 0.32]	
S2	Europe	**0.94 [0.93, 0.95]**	**0.95 [0.92, 0.97]**	**0.94 [0.92, 0.96]**	
S3	South America and unsampled source population	0.00 [0.00, 0.01]	0.00 [0.00, 0.00]	0.00 [0.00, 0.30]	
S4	Europe and unsampled source population	0.04 [0.03, 0.05]	0.04 [0.02, 0.07]	0.05 [0.00, 0.34]	

Before comparing possible scenarios, we confirmed that at least one combination of scenarios and priors can produce simulated data sets that are close enough to the observed data using the pre-evaluation of scenarios and prior distributions option. This was performed through a principal component analysis (Supporting information S1a to S7a). Once the best scenario (S2) was selected according to the logistic regression test (Supporting information S1b to S7b), the discrepancy between the model-posterior combination and the observed data was evaluated for each target population. Although we obtained many summary statistics on the tails of distributions, all the seven principal component analyses (PCA) showed that posterior values were close to the observed dataset (Supporting information S1c to S7c). We concluded that the adequacy of the model-posterior combination sufficed to correctly explain the observed dataset.

## Discussion

Because it is not always possible to compare/merge microsatellite data produced by different laboratories, due to subtle differences in the protocols used^31^, we decided to collect and genotype, using a fully standardized protocol, 22 populations of the potato cyst nematode *G. rostochiensis* originating from all inhabited continents. Our results confirmed the South American origin for this species and its initial introduction into Europe. Moreover, our analyses provided support for the hypothesis that populations from North America, Africa, Asia and Oceania most likely originated from Europe. This suggests that Europe served as a bridgehead in the worldwide invasion history of *G. rostochiensis*.

Among the 22 genotyped *G. rostochiensis* populations, only two (HAR2 from Kenya and Jap-Ho from Japan) showed a heterozygote deficit (*F*_IS_ > 0). This finding is surprising in the context of cyst nematodes, as heterozygote deficits were frequently found in populations of *G. pallida*^4^, *Heterodera schachtii*^5,33^, *H. glycines*^34^ and *H. carotae*^35,36^. The heterozygote deficit in cyst nematodes was attributed to the low active dispersal ability of juveniles leading to inbreeding^37^. Future research is needed to explain this particular genetic feature of *G. rostochiensis* populations, which suggests the possibility of a significant cost of consanguineous mating or capacity of the females of this species to preferentially mate with non-sibling males.

In species introduction events, the level of genetic diversity allows distinguishing source and sink populations: an introduced population being less diverse than a native one due to the bottleneck effect resulting from the introduction of a limited number of individuals. Our results confirm that the potato cyst nematode *G. rostochiensis* is originated from South America^8,38,39^, as the four *G. rostochiensis* populations from South America were the most genetically diverse (H_nb_ > 0.235; Table 1). All the other populations were introduced/sink populations, but the less genetically diverse populations could originate either from South America or from Europe, as the European populations showed an intermediate level of genetic diversity (Table 1).

Based on the matrix of pairwise *F*_ST_ (Fig. 1) and the genetic clustering output (Fig. 2), we can hypothesise that Indonesian, Kenyan and Australian *G. rostochiensis* populations, which were characterized by a strong genetic homogeneity and low *F*_ST_ among populations from the same geographic area, were probably originated respectively from a single introduction event and subsequently spread within each respective country. Moreover, the *G. rostochiensis* populations from North America were shown to belong to two distinct genetic clusters, confirming that the nematode was introduced twice into this continent^24^. The same pattern was observed in Asia, with at least two distinct introduction events as all four Indonesian populations were assigned to cluster 2 while the remaining Asian populations (Jap-Ho from Japan and Leb-Be from Lebanon) were assigned to the genetic cluster 1 (Fig. 2).

The global distribution of the nematode leads to a large number of possible evolutionary scenarios among *G. rostochiensis* populations. To reduce the number of scenarios to be tested, we grouped populations according to their geographical origins and pre-existing historical knowledge. The European populations were unintentionally introduced from South America, after the mid-nineteenth century, due to the importation of potato tubers^8,9^. Since this period, and due to increased trade between Europe and the rest of the world, Europe likely became a second significant source for the dispersion of *G. rostochiensis* worldwide. Consequently, South American populations (Bolivia, Peru and Chile) were grouped together to represent the native area of *G. rostochiensis*, as well as European populations to represent a likely second significant region of origin for its worldwide dispersion. Due to low *F*_ST_ values observed among populations from the same country, the four Kenyan populations were pooled together, as well as the four Indonesian populations and the two Australian populations. This allowed us to elucidate the invasion history of *G. rostochiensis* by evaluating two alternative hypotheses for each target population: the population was introduced from South America, the centre of origin of PCN, or from Europe after its initial establishment into this continent. Our results clearly showed that all the target populations, collected in Canada, the USA, Kenya, Indonesia, Lebanon, Japan and Australia, were all introduced from Europe (Table 2 and Supporting information). Means of transportation of the nematode are largely unknown and could be very diverse. In the case of introduction from South America to Europe it is highly possible that nematode cysts were distributed with the imported potato tubers from South America^9,38^. Spears^38^ also suggested that in the case of the introduction in the USA, military equipment brought back from Europe after World War I may have been a possible means of nematode invasion. Our results support the likelihood of such a scenario, although we still lack specific evidence about the actual means of nematode transportation. Clearly other means of long-distance transportation exist, including contaminated soil within shipments of bulbs, ornamental plants, etc.^40^. In Japan, an unexpected pathway was discovered through imported Peruvian guano contaminated with viable nematode cysts and subsequently used as a fertilizer^41^. This discovery led the authors to suggest that *G. rostochiensis* was introduced to Japan from Peru. Our results, pointing to a European origin, suggested that various transportation means might have occurred in Japan. Since we have only examined one Japanese population here, further research is necessary to investigate the genetic diversity among different *G. rostochiensis* populations in Japan.

A strong bottleneck occurred from South America to Europe, leading to much lower genetic diversity in Europe than in the native area of *G. rostochiensis*, and Europe subsequently served as the source for waves of invasions worldwide. The important role of Europe in worldwide dispersal has been reported for many species, pathogenic or not. For instance, the grapevine downy mildew, *Plasmopara viticola*, which originated in North America, was first introduced into Europe from where it further invaded different continents^42^. Furthermore, the mealybug *Pseudococcus viburni*, an insect infecting a large number of plant families, had a similar mode of global spread to that of *G. rostochiensis*: Europe was initially invaded from South America and later became the main source of worldwide spread^43^. Generally, the bridgehead invasion scenario, where an introduced population serves as the source of other invasive populations, applies to many cases of agricultural pest introductions^44^.

The low *F*_ST_ measured among *G. rostochiensis* populations from the same geographical area (Indonesia, Kenya and Australia) suggested that in each country these populations recently diverged from their common ancestor (a recent introduction event) or that gene flow still remain strong between these populations in each country. The latter is less probable due to the low dispersal abilities of nematodes and the strict regulatory measures applied in many countries. Although introductions in Indonesia, Kenya and Australia occurred more recently compared to that in Europe, we cannot determine the timing of introduction events for the populations Ama, US, Leb-Be and Jap-Ho populations since we have genotyped only one population in these countries. However, the different dates of first detection also suggested that the introduction was more recent than the initial one in Europe. ABC analyses could have allowed us to estimate the number of generations since the introduction event. However, due to the variations in the collection times of different populations and the variable number of generations per year for *G. rostochiensis* based on environmental conditions, we were unable to perform this type of estimation accurately.

Quarantine status of some plant pests is part of crop protection strategies and has generally not received the scientific support it deserves. Our results support the idea that the quarantine status of *G. rostochiensis*, a pathogen with a narrow host range, has effectively prevented its further spread from South America to countries outside Europe. Using sequences of mitochondrial genes, Subbotin et al.^45^ suggested that the centre of origin for *G. rostochiensis* could be in the south of Bolivia or north-west Argentina. Our results, which show that Bolivian populations are the most diverse and the only ones assigned to cluster 3, confirm this putative centre of origin and may be used to guide quarantine regulations in efforts to prevent further invasions by genetically distant and diverse *G. rostochiensis* populations. New invasions from its native area could significantly increase diversity and facilitate nematode adaptation to chemical nematicides and resistant potato cultivars (e.g.,^46^). Regarding the last recent introductions, we cannot rule out the possibility that in some cases the introduction occurred before the implementation of quarantine measures in the country. Moreover, each introduction of an exotic pathogen is not always successful due to the absence of suitable establishment conditions. But, as the frequency of introduction events increases, the likelihood of getting the right conditions also rises. As growers worldwide seek for the best cultivars to compete globally, the international transport of seed tubers is expected to increase. Importing material from approved suppliers with processes to minimize pests and diseases incidence in seed tuber fields could be an effective way to further reduce the risk of new introductions in addition to the quarantine status and sole proof of pest absence in the field of origin of the plant material.

## Conclusion

Microsatellite-based genotyping of *Globodera rostochiensis* populations from all continents allowed for a reconstruction of invasion routes at a worldwide scale. This collaborative population genetic study confirms that this plant-parasitic nematode species evolved in the Andean region of South America and was first introduced into Europe. Genetically less diverse populations sampled in North America, Africa, Asia and Oceania, most likely originate from Europe, which served as a secondary distribution hub for the worldwide invasion of *G. rostochiensis*. These insights are not only essential to prevent further introductions of genetically diverse populations but also to enable the development of effective control strategies. Control methods proven successful within the European secondary hub could be extrapolated to effectively manage *G. rostochiensis* populations in other global regions stemming from this hub.

## Methods

### *Globodera rostochiensis* populations

In this study, 22 *G. rostochiensis* populations collected from different parts of the world (Supporting information Table S1) were genotyped using microsatellite markers. Seventeen populations were selected based on four previously published studies, and five new populations were added. We selected seven populations among the 15 used by Boucher et al.^24^: populations B2 and B4 from Bolivia, 267 from Peru, 3346 from Chile, Ama from Canada, Dunk from France, and Port from Portugal. Two Australian populations were chosen and obtained from Blacket et al.^26^: Cora Lynn (CL) and Thorpdale (TH). Four Indonesian *G. rostochiensis* populations were also chosen and obtained from Handayani et al.^27^: two from North Sumatra (NRK-4 and NRK-6) and two from East Java (NRM-1 and NRM-2). In Kenya, we obtained four populations from Mwangi et al.^25^: three from Nyandarua (HAR2, KIN2, RIR) and one from Kiambu (TGN). Finally, the remaining five new populations of the dataset included: a Lebanese population (Leb-Be), a Czech population (CZ), a Dutch population (NL), a North American population from New York state (US), and a Japanese population from Hokkaido (Jap-Ho).

### Microsatellite genotyping

Total genomic DNA was extracted from single individual juveniles as described by Boucher et al.^24^. For each population, one second-stage juvenile (J2) was isolated per cyst and a total of 35–50 J2s from 35–50 distinct cysts were used for DNA extraction. DNA quality was validated by PCR amplification of an ITS fragment according to Thiéry and Mugniéry^47^. DNA samples showing a positive amplification of the ITS marker were then processed for microsatellite PCR amplification and genotyping. A set of 11 microsatellite markers developed by Boucher et al.^24^ was used in three multiplex combinations. The marker Gp116 used by Boucher et al.^24^ was discarded because of the presence of a second microsatellite motif in the sequence^26^. All the primers were synthetized at Thermo Fisher Scientific. The panel 1 included Gr50 (6FAM), Gp109 (NED), Gp126 (PET) and Gp135 (VIC). The panel 2 included Gr85 (VIC), Gr96 (6FAM) and Gp118 (PET). The panel 3 included Gr67 (6FAM), Gr75 (NED), Gr90 (VIC) and Gr91 (PET). PCR reagents, volume and cycling conditions used were as described by Boucher et al.^24^. PCR multiplex were performed on a 96-well Thermal Cycler (Applied Biosystems). PCR products were then diluted to 1:40 in sterile water, and 3 μL of this dilution was mixed with 0.05 μL of GeneScan 500 LIZ Size Standard (Applied Biosystems) and 5 μL of formamide (Applied Biosystems). Genotyping was performed on ABI 3730XL sequencer (Applied Biosystems) at the Gentyane INRAE platform. Allele sizes were identified using the automatic calling and binning procedure of GeneMapper^®^ v 5.0 (Thermo Fisher Scientific) and completed by a manual examination.

### Analyses of population genetic structure and diversity

To explore the genetic diversity and structure of the 22 *G. rostochiensis* populations, 840 individual juveniles were genotyped. Data analyses were done on a reduced dataset free of any missing data (i.e., on 793 individuals). Allelic richness (Ar) was estimated using the rarefaction method implemented in POPULATIONS 1.2.32^48^, which estimated the mean number of alleles per locus for a reduced sample size. An unbiased estimate of gene diversity (H_nb_ according to Nei^49^) and deviation from random mating (*F*_IS_) were computed using GENETIX 4.05.2^50^. The statistical significances of *F*_IS_ were estimated using the allelic permutation method (10,000 permutations) implemented in GENETIX.

The differentiation coefficients between each pair of populations (*F*_ST_) were computed using GENEPOP 4.5.1 according to Weir and Cockerham^51^, and their statistical significances were estimated by 5000 random permutations of individuals among populations. A Bonferroni adjustment was applied to take into account multiple testing, i.e., α = 0.05 was lowered to α = 0.00022 for 231 comparisons (22 × 21/2).

Genetic structure of the 22 *G. rostochiensis* populations was explored using the Bayesian clustering algorithms implemented in STRUCTURE 2.3.4^52,53^. For each number of genetic clusters K (from 1 to 22), ten independent runs were executed using the admixture model, uncorrelated allele frequency and the default priors except for alpha, for which the value was set to 0.0454 (i.e., 1/p, p being the number of populations) following the recommendations of Wang^54^. The initial burn-in period consisted of 1,000,000 iterations and the number of Markov Chain Monte Carlo (MCMC) repetitions was 3,000,000. Structure Harvester Web ver.0.6.94^55^ was applied to determine the most likely number of clusters statistically determined using the ad-hoc Evanno statistic ΔK^56^. The ten independent replicates for the optimal value of K were then merged using CLUMPP version 1.1.2^57^.

### Inferences of global invasion history

To investigate the most likely pathways for establishment of *G. rostochiensis* populations in the different parts of the world, Approximate Bayesian Computation (ABC) methods implemented in DIYABC 2.1.0^58^ were used. Based on historical knowledge of the origins of *G. rostochiensis*, we excluded the hypothesis that some populations were native except for the South American populations (Bolivia, Peru and Chile) and we draw for each target population (Canada, United States, Kenya, Indonesia, Lebanon, Japan and Australia) two origins and main route hypotheses. The first hypothesis is a direct South American origin, the second hypothesis is an origin from Europe after the initial introduction into this continent.

To reduce the number of scenarios, we grouped populations into pools. Five pools of populations were constituted: South America (populations B2, B4, 267 and 3346), Europe (populations Dunk, Port, NL and CZ), Kenya (populations HAR2, KIN2, TGN and RIR), Indonesia (populations NRK4, NRK6, NRM2 and NRM1) and Australia (populations CL and TH). It is noteworthy that North American populations Ama (Canada) and US (USA) were not pooled together because the results of Boucher et al.^24^ showed that they were probably resulting from two distinct introduction events in North America.

For each of the seven target populations, Ama (Canada, North America), US (USA, North America), Kenya (East Africa), Indonesia (Southeast Asia), Leb-Be (Lebanon, West Asia), Jap-Ho (Japan, East Asia) and Australia (Oceania), four scenarios were constructed reflecting its possible origin. Scenarios S1 and S3 tested a direct South American origin while the European origin was tested by scenarios S2 and S4. All scenarios were constrained by the fact that the oldest demographic event corresponds to the split between South America and Europe. Both possible origins were tested in a tree topology integrating (scenarios S3 and S4) or not integrating (scenarios S1 and S2) the effect of a possible unsampled source population (i.e., a ghost population), itself introduced from South America or from Europe. Divergence events were followed by a bottleneck representing the signal of an introduction event (Fig. 3).

We formalized the four scenarios, prior distributions and computed summary statistics for each target unit. Seven reference tables were built under the Generalized Stepwise Mutation model with (i) mean mutation rate ranging from 0.0001 to 0.001 and uniform prior distribution, (ii) mean parameter of the geometric distribution (mean P) ranging from 0.1 to 0.3 and uniform prior distribution and (iii) t2 > t1 and t3 > t2 (Fig. 3). Three single sample statistics were computed: the mean number of alleles per locus, the mean gene diversity across loci^49^ and the mean allele size variance across loci. For each pair of populations, the pairwise *F*_ST_ values^51^, the mean index of classification^59,60^ and the distance between two samples^61^ were computed. 4,000,000 simulations datasets, i.e., 1,000,000 simulations datasets per scenario, were run.

Possible scenarios were compared through the computation of the posterior probabilities of each scenario. The relative posterior probabilities of the competing scenarios were assessed by a logistic regression estimate. We also used the option ‘model checking’ with PCA in DIYABC, using all summary statistics, to evaluate how well the best scenario and parameter posterior distributions combination fit the observed data.

### Additional information

The different *Globodera rostochiensis* populations were collected by the different co-authors who obtained the required authorizations. They provided to INRAE the biological material following official endorsed Letter Of Authorization necessary for shipment of quarantine organisms between laboratories. The INRAE laboratory is accredited to hold and perform experiments on this quarantine organism. Authors declare that the cyst nematode species, *Globodera rostochiensis*, sampled in the present work is not concerned by both the IUCN Policy Statement on Research Involving Species at Risk of Extinction and the Convention on the Trade in Endangered Species of Wild Fauna and Flora.

### Supplementary Information

Supplementary Information.

## Data Availability

The file (Esquibet_Gros.txt) containing the genotypic data (Genepop format) for the 22 *Globodera rostochiensis* populations is available at data.inrae.fr (10.57745/3INH2O).

## References

[1] Elling AA (2013). Major emerging problems with minor *Meloidogyne* species. Phytopathology.

[2] Jones JT (2013). Top 10 plant-parasitic nematodes in molecular plant pathology. Mol. Plant Pathol..

[3] Wallace HR (1968). The dynamics of nematode movement. Annu. Rev. Phytopathol..

[4] Picard D, Plantard O, Scurrah M, Mugniéry D (2004). Inbreeding and population structure of the potato cyst nematode (*Globodera pallida*) in its native area (Peru). Mol. Ecol..

[5] Plantard O, Porte C (2004). Population genetic structure of the sugar beet cyst nematode *Heterodera schachtii*: A gonochoristic and amphimictic species with highly inbred but weakly differentiated populations. Mol. Ecol..

[6] Alenda C, Montarry J, Grenier E (2014). Human influence on the dispersal and genetic structure of French *Globodera tabacum* populations. Infect. Genet. Evol..

[7] Silvestre R, Dandurand LM, Zasada IA, Franco J, Kuhl JC (2021). An assessment of potato cyst nematode (*Globodera* spp.) research from the Andean region of South America. Part 1: Occurrence and impact. Nematropica.

[8] Evans K, Franco J, De Scurrah MM (1975). Distribution of species of potato cyst-nematodes in South America. Nematologica.

[9] Grenier E, Fournet S, Petit E, Anthoine G (2010). A cyst nematode ‘species factory’ called the Andes. Nematology.

[10] Cannon OS (1941). *Heterodera schachtii* found in a Long Island potato field. Plant Dis. Rep..

[11] Olsenand OA, Mulvey RH (1962). The discovery of golden nematode in Newfoundland. Can. Plant Dis. Surv..

[12] Mahran A (2010). The golden potato cyst nematode *Globodera rostochiensis* pathotype Ro1 in the Saint-Amable regulated area in Quebec, Canada. Plant Dis..

[13] Yamada E, Takakura S, Tezuka H (1972). On the occurrence of the potato cyst-nematode, *Heterodera rostochiensis* Wollenweber, in Hokkaido, Japan. Jpn. J. Nematol..

[14] Ibrahim SK, Saad AT, Haydock PPJ, Al-Masri Y (2000). Occurrence of the potato cyst nematode *Globodera rostochiensis* in Lebanon. Nematology.

[15] Jiang R (2022). First record of the golden potato nematode *Globodera rostochiensis* in Yunnan and Sichuan provinces of China. J. Integr. Agric..

[16] Stanton JM (1986). First record of potato cyst nematode, *Globodera rostochiensis*, in Australia. Australas. Plant Pathol..

[17] Collins SJ, Marshall JM, Zhang XH, Vanstone VA (2010). Area freedom from *Globodera rostochiensis* in Western Australia. Asp. Appl. Biol..

[18] Guy G, Woodward J, Hinch JM (1992). *Globodera*
*rostochiensis* and possibly *G*. *pallida* in Australia. J. Nematol..

[19] Mulyadi M, Rahayu B, Triman B, Indarti S (2003). Identification of golden potato cyst nematode (*Globodera rostochiensis*) in Batu, East Java. J. Perlind. Tanam. Indones..

[20] Kleynhans KPN, Marks RJ, Brodie BB (1998). Potato cyst nematodes (*Globodera* species) in Africa. Potato Cyst Nematodes. Biology, Distribution and Control.

[21] Mwangi JM, Kariuki GM, Waceke JW, Grundler FM (2015). First report of *Globodera rostochiensis* infesting potatoes in Kenya. New Dis. Rep..

[22] Niragire I, Couvreur M, Karssen G, Uwumukiza B, Bert W (2019). First report of potato cyst nematode (*Globodera*
*rostochiensis*) infecting potato (*Solanum*
*tuberosum* L.) in Rwanda. Plant Dis..

[23] Cortada L (2020). First report of potato cyst Nematode, *Globodera rostochiensis*, infecting potato (*Solanum tuberosum*) in Uganda. Plant Dis..

[24] Boucher AC (2013). Genetic diversity of the golden potato cyst nematode *Globodera rostochiensis* and determination of the origin of populations in Quebec, Canada. Mol. Phylogenet. Evol..

[25] Mwangi, J. M. Resistance based integrated pest management strategy for *Globodera rostochiensis* and *Globodera pallida* in potato cropping systems. PhD Thesis, University of Kassel. 10.17170/kobra-20191212863 (2019).

[26] Blacket MJ (2019). Molecular assessment of the introduction and spread of potato cyst nematode, *Globodera rostochiensis*, in Victoria, Australia. Phytopathology.

[27] Handayani ND (2020). Distribution, DNA barcoding and genetic diversity of potato cyst nematodes in Indonesia. Eur. J. Plant Pathol..

[28] Handayani ND (2022). Genomic reconstruction of the introduction and diversification of golden potato cyst nematode populations in Indonesia. Phytopathology.

[29] Delmotte F, Leterme N, Simon JC (2001). Microsatellite allele sizing: Difference between automated capillary electrophoresis and manual technique. BioTechniques.

[30] Hahn M, Wilhelm J, Pingoud A (2001). Influence of fluorophore dye labels on the migration behaviour of polymerase chain reaction—Amplified short tandem repeats during denaturing capillary electrophoresis. Electrophoresis.

[31] Vignal A, Milan D, San Cristobal M, Eggen A (2002). A review on SNP and other types of molecular markers and their use in animal genetics. Genet. Sel. Evol..

[32] Selkoe AK, Toonen RJ (2006). Microsatellites for ecologists: A practical guide to using and evaluating microsatellite markers. Ecol. Lett..

[33] Kim J (2019). Phylogeography of the highly invasive sugar beet nematode, *Heterodera schachtii* (Schmidt, 1871), based on microsatellites. Evol. Appl..

[34] Wang HM, Zhao HH, Chu D (2015). Genetic structure analysis of populations of the soybean cyst nematode, *Heterodera glycines*, from north China. Nematology.

[35] Gautier C (2019). Microsatellite markers reveal two genetic groups in European populations of the carrot cyst nematode *Heterodera carotae*. Infect. Genet. Evol..

[36] Esquibet M (2020). Evidence of strong gene flow among French populations of the carrot cyst nematode *Heterodera carotae*. Plant Pathol..

[37] Montarry J (2015). Heterozygote deficits in cyst plant-parasitic nematodes: Possible causes and consequences. Mol. Ecol..

[38] Spears, J. F. The golden nematode. Handbook. United States Department of Agriculture, Agriculture Handbook No. 353 (1968).

[39] Evans K, Stone AR (1977). A review of the distribution and biology of the potato cyst-nematodes *Globodera*
*rostochiensis* and *G*. *pallida*. Trop. Pest Manag..

[40] Steiner G, Taylor AL, Cobb GS (1951). Cyst forming plant parasitic nematodes and their spread in commerce. Proc. Helm. Soc. Wash..

[41] Inagaki H, Kegasawa K (1973). Discovery of the potato cyst nematode, *Heterodera rostochiensis* Wollenweber, 1923, (Tylenchida: Heteroderidae) from Peru guano. Appl. Entomol. Zool..

[42] Fontaine MC (2021). Europe as a bridgehead in the worldwide invasion history of the grapevine downy mildew, *Plasmopara*
*viticola*. Curr. Biol..

[43] Correa MCG (2019). European bridgehead effect in the worldwide invasion of the obscure mealybug. Biol. Invas..

[44] Guillemaud T, Ciosi M, Lombaert E, Estoup A (2011). Biological invasions in agricultural settings: Insights from evolutionary biology and population genetics. C. R. Biol..

[45] Subbotin SA, Franco J, Knoetze R, Roubtsova TV, Bostock RM, Cid del Prado Vera I (2020). DNA barcoding, phylogeny and phylogeography of cyst nematode species from the genus *Globodera* (Tylenchida: Heteroderidae). Nematology.

[46] McDonald BA, Linde C (2002). Pathogen population genetics, evolutionary potential, and durable resistance. Annu. Rev. Phytopathol..

[47] Thiéry M, Mugniery D (1996). Interspecific rDNA restriction fragment length polymorphism in *Globodera* species, parasites of Solanaceous plants. Fundam. Appl. Nematol..

[48] Langella, O. Populations 1.2.31. Population Genetic Software (Individuals or Populations Distances, Phylogenetic Trees) 2012. http://bioinformatics.org/~tryphon/populations/ (1999).

[49] Nei M (1978). Estimation of average heterozygosity and genetic distance from a small number of individuals. Genetics.

[50] Belkhir K, Borsa P, Chikhi L, Raufaste N, Bonhomme F (2004). GENETIX 405, logiciel sous Windows™ pour la génétique des populations.

[51] Weir BS, Cockerham CC (1984). Estimating F-statistics for the analysis of population structure. Evolution.

[52] Pritchard JK, Stephens M, Donnelly P (2000). Inference of population structure using multilocus genotype data. Genetics.

[53] Falush D, Stephens M, Pritchard JK (2003). Inference of population structure using multilocus genotype data: Linked loci and correlated allele frequencies. Genetics.

[54] Wang J (2017). The computer program structure for assigning individuals to populations: Easy to use but easier to misuse. Mol. Ecol. Resour..

[55] Earl DA, von Holdt BM (2012). STRUCTURE HARVESTER: A website and program for visualizing STRUCTURE output and implementing the Evanno method. Conserv. Genet. Resour..

[56] Evanno G, Regnaut S, Goudet J (2005). Detecting the number of clusters of individuals using the software STRUCTURE: A simulation study. Mol. Ecol..

[57] Jakobsson M, Rosenberg NA (2007). CLUMPP: A cluster matching and permutation program for dealing with label switching and multimodality in analysis of population structure. Bioinformatics.

[58] Cornuet JM (2014). DIYABC v2.0: A software to make approximate Bayesian computation inferences about population history using single nucleotide polymorphism, DNA sequence and microsatellite data. Bioinformatics.

[59] Rannala B, Mountain JL (1997). Detecting immigration by using multilocus genotypes. Proc. Natl. Acad. Sci. USA.

[60] Pascual M (2007). Introduction history of *Drosophila subobscura* in the New World: A microsatellite based survey using ABC methods. Mol. Ecol..

[61] Goldstein DB, Linares AR, Cavalli-Sforza LL, Feldman MW (1995). An evaluation of genetic distances for use with microsatellite loci. Genetics.

