# Supplementary material for: Europe as a secondary distribution hub in the worldwide invasion of the potato cyst nematode Globodera rostochiensis
Source: Sci Rep. 2024 Jun 17;14:13915. doi: 10.1038/s41598-024-64617-0 (PMC11183074; doi:10.1038/s41598-024-64617-0)
Supplement: Supplementary file 1 — Supplementary Information. [file 41598_2024_64617_MOESM1_ESM.docx]

**Europe as a secondary distribution hub in the worldwide invasion of the potato cyst nematode *Globodera rostochiensis***

Magali ESQUIBET^1^, James M. MWANGI^2,3^, Sebastian KIEWNICK^3^, Xiaohong WANG^4^, Benjamin MIMEE^5^, Nurul Dwi HANDAYANI^6,11^, Wim BERT^6^, Johannes HELDER^7^, John WAINER^8^, Itaru SAKATA^9^, Nathan GARCIA^10^, Eric GRENIER^1^ and Josselin MONTARRY^1*^

^1^ IGEPP, INRAE, Institut Agro, Univ Rennes, Le Rheu, France.

^2^ Department of Biological Sciences, Chuka University, Kenya.

^3^ Julius Kühn-Institut, 38104 Braunschweig, Germany.

^4^ US Department of Agriculture, Agricultural Research Service, Robert W. Holley Center for Agriculture and Health and School of Integrative Plant Science, Cornell University, Ithaca, NY, USA.

^5^ Agriculture and Agri-Food Canada, Saint-Jean-sur-Richelieu, Québec, Canada.

^6^ Nematology Research Unit, Department of Biology, Ghent University, Ghent, Belgium.

^7^ Laboratory of Nematology, Wageningen University, The Netherlands.

^8^ AgriBio Centre, Agriculture Victoria Research, Melbourne, Australia.

^9^ Hokkaido Agricultural Research Centre (HARC), National Agriculture and Food Research Organization (NARO), Hokkaido, Japan.

^10^ ANSES, Plant Health Laboratory, Nematology Unit, F-35653 Le Rheu, France.

^11^ Indonesian Agricultural Quarantine Agency, Ministry of Agriculture, E Building 5^th^ Floor, Jl. Harsono RM. 3 Ragunan, Jakarta 12550, Indonesia.

^*^ Corresponding author: josselin.montarry@inrae.fr

**Table S1:** Details about the 22 *Globodera rostochiensis* populations used in this study. Cysts of the different populations came from soil samples collected in potato fields. The GPS location was not available for several populations (NA).

| **Code** | **Continent** | **Country** | **Province** | **Closest city** | **GPS location** |
| --- | --- | --- | --- | --- | --- |
| B2 | South-America | Bolivia | Jose Maria Linares | Capaña | NA |
| B4 | South-America | Bolivia | Cochabamba | Tiraque | NA |
| 267 | South-America | Peru | Puno | Huancane | -15.3463889 -69.985722 |
| 3346 | South-America | Chile | Coquimbo | La Serena | NA |
| CZ | Europe | Czech Republic | Plzeň | Svojše | NA |
| Dunk | Europe | France | Hauts-de-France | Dunkerque | NA |
| NL | Europe | The Netherlands | Gueldre | Wageningen | NA |
| Port | Europe | Portugal | Norte | Montalegre | NA |
| Ama | North-America | Canada | Quebec | St-Amable | NA |
| US | North-America | United States | New-York State | Steuben county | NA |
| HAR2 | Africa | Kenya | Nyandarua | Haraka | -0.77588 36.61652 |
| KIN2 | Africa | Kenya | Nyandarua | Kinangop | -0.58854 36.61234 |
| TGN | Africa | Kenya | Kiambu | Tigoni | -1.15184 36.68523 |
| RIR | Africa | Kenya | Nyandarua | Rironi | -0.3150195 36.48329 |
| NRK4 | Asia | Indonesia, Sumatra | Karo | Lingga Julu | 3.13555 98.47249 |
| NRK6 | Asia | Indonesia, Sumatra | Karo | Suka Ndebi | 3.19888 98.47500 |
| NRM1 | Asia | Indonesia, Java | Batu | Sumber Brantas | -7.75328 112.53643 |
| NRM2 | Asia | Indonesia, Java | Batu | Krajan | -7.86503 112.55813 |
| Leb-Be | Asia | Lebanon | Beyrouth | Beyrouth | NA |
| Jap-Ho | Asia | Japan | Hokkaido | Kutchan | NA |
| CL | Oceania | Australia | Victoria | Cora Lynn | -38.14 145.61 |
| TH | Oceania | Australia | Victoria | Thorpdale | -38.3 146.1 |

**
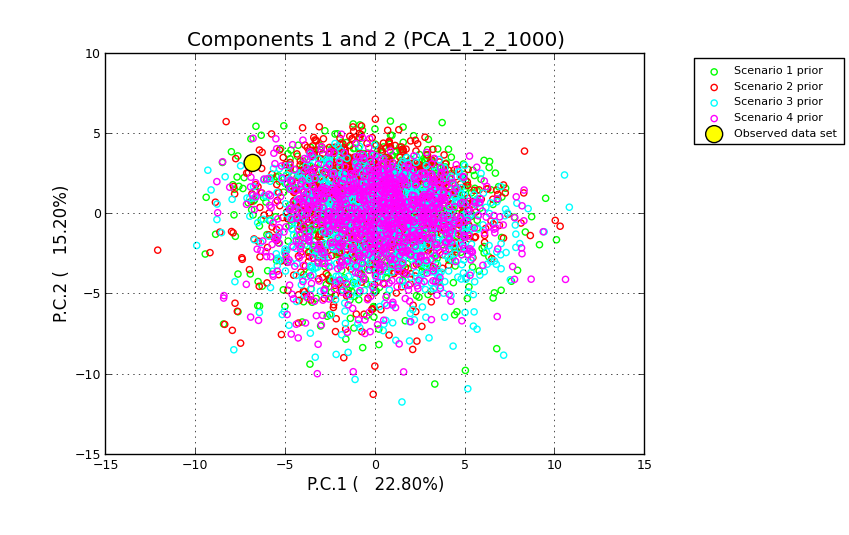
(a)**

**
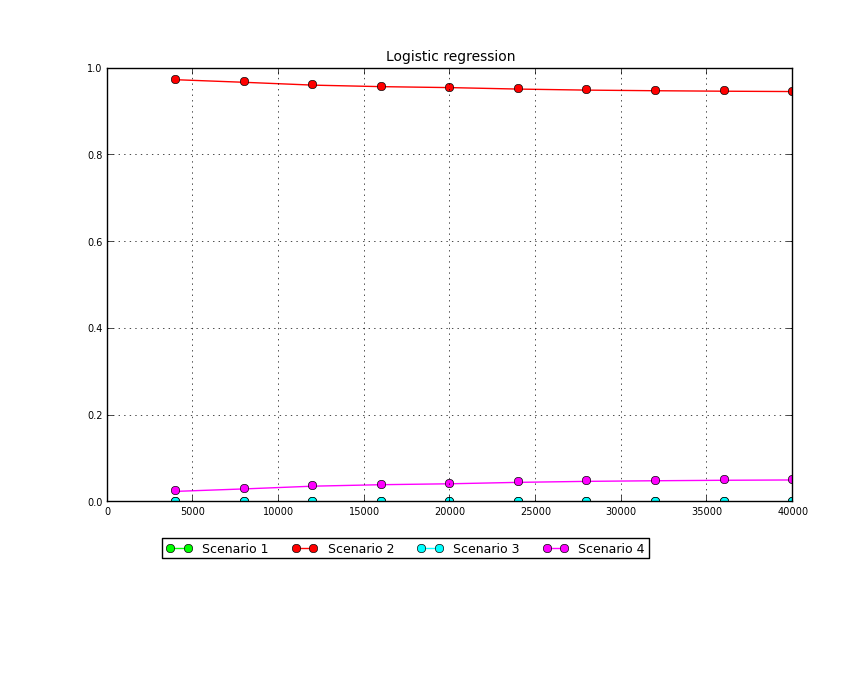
(b)**

**(c)**


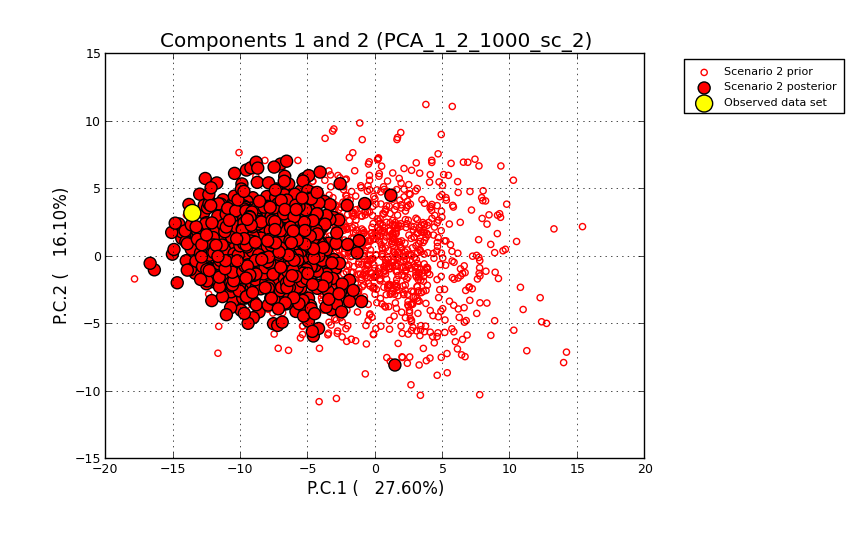


**Fig. S1** Results of the DIYABC analyses for the Canadian population (Ama).

**(a):** principal component analysis (PCA) to check that at least one combination of scenarios and priors can produce simulated data sets that are close enough to the observed data; **(b):** model comparison based on a logistic regression approach; **(c):** model checking with PCA to evaluate how well the best scenario and parameter posterior distribution combinations fit the observed data.

**
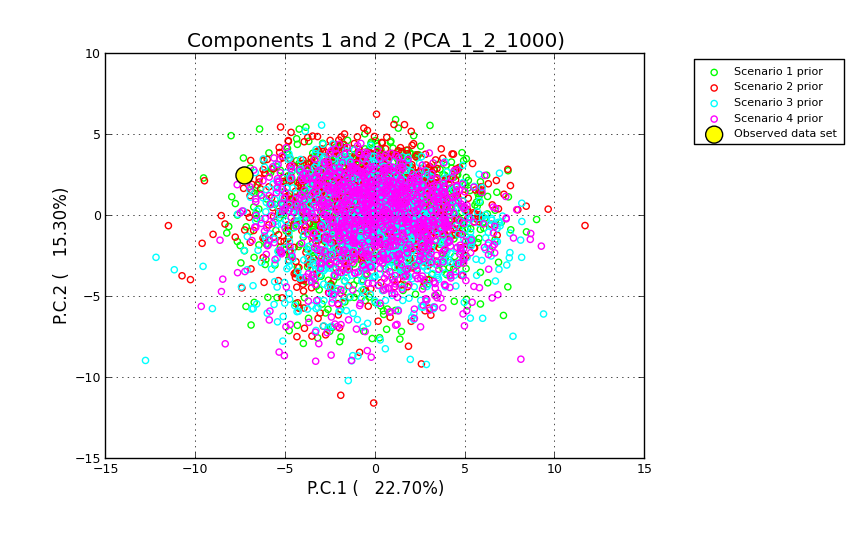
(a)**

**
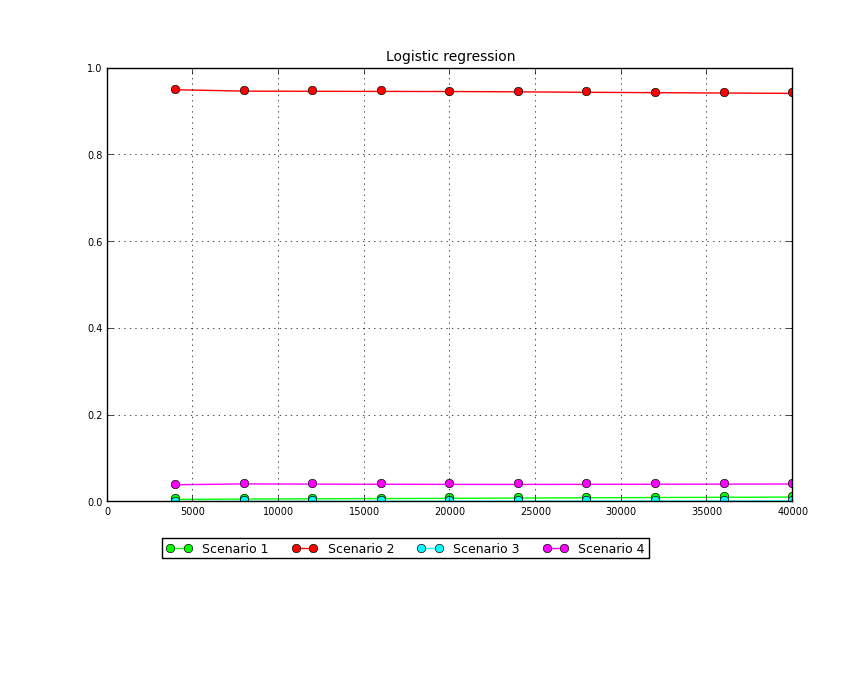
(b)**

**
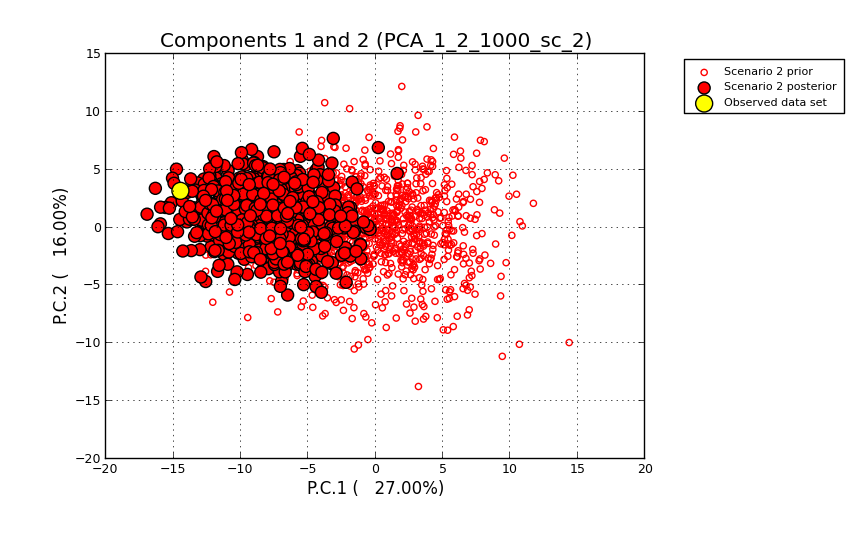
(c)**

**Fig. S2** Results of the DIYABC analyses for the American population (US).

**(a):** principal component analysis (PCA) to check that at least one combination of scenarios and priors can produce simulated data sets that are close enough to the observed data; **(b):** model comparison based on a logistic regression approach; **(c):** model checking with PCA to evaluate how well the best scenario and parameter posterior distribution combinations fit the observed data.

**(a)**


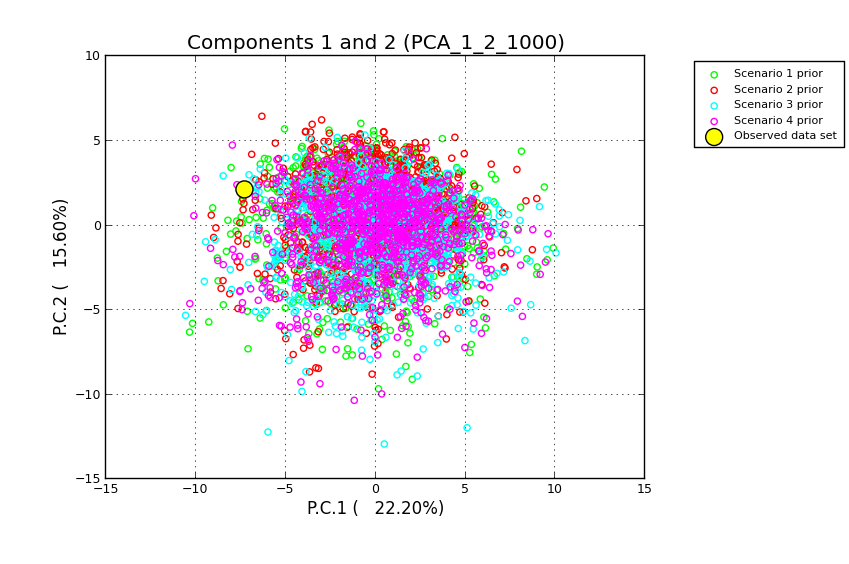


**(b)**


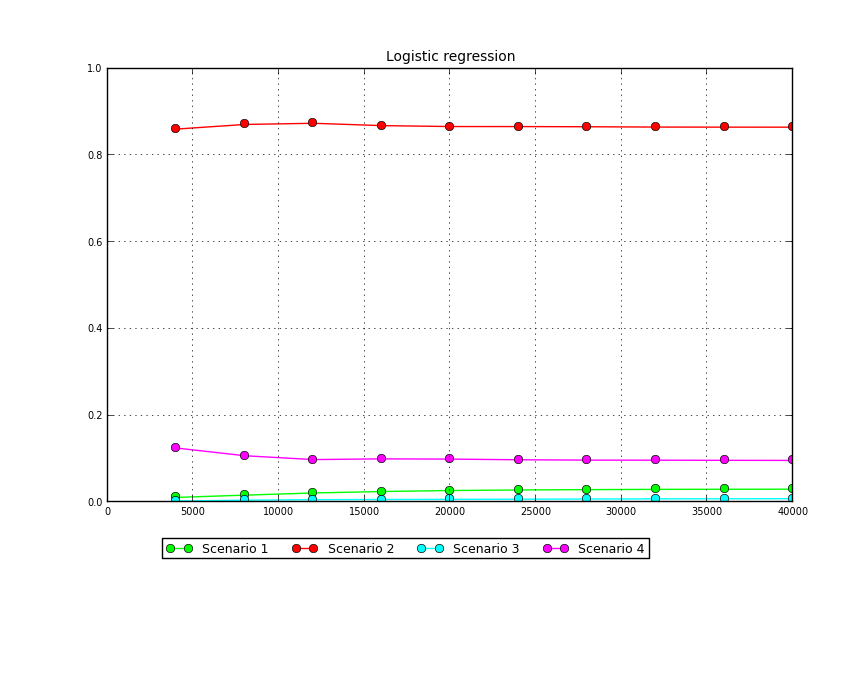


**(c)**


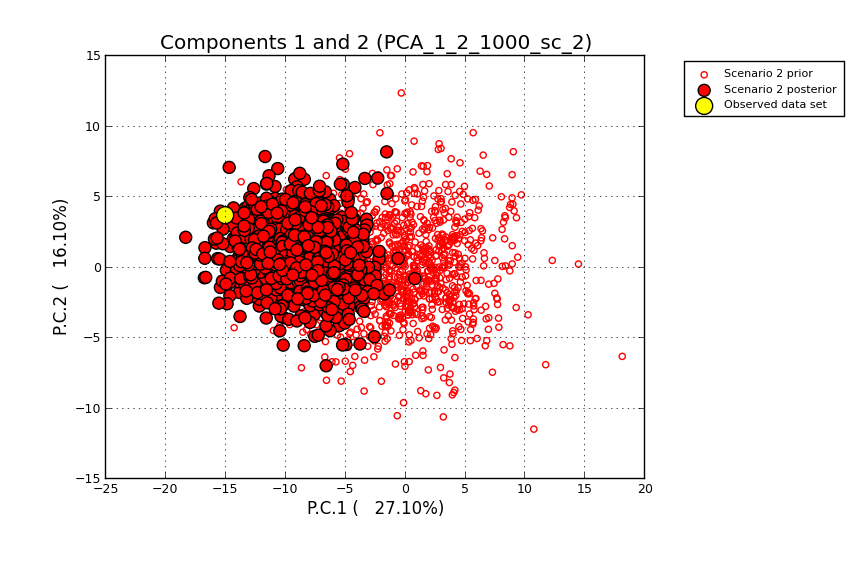


**Fig. S3** Results of the DIYABC analyses for the East African populations (Kenya).

**(a):** principal component analysis (PCA) to check that at least one combination of scenarios and priors can produce simulated data sets that are close enough to the observed data; **(b):** model comparison based on a logistic regression approach; **(c):** model checking with PCA to evaluate how well the best scenario and parameter posterior distribution combinations fit the observed data.

**(a)**


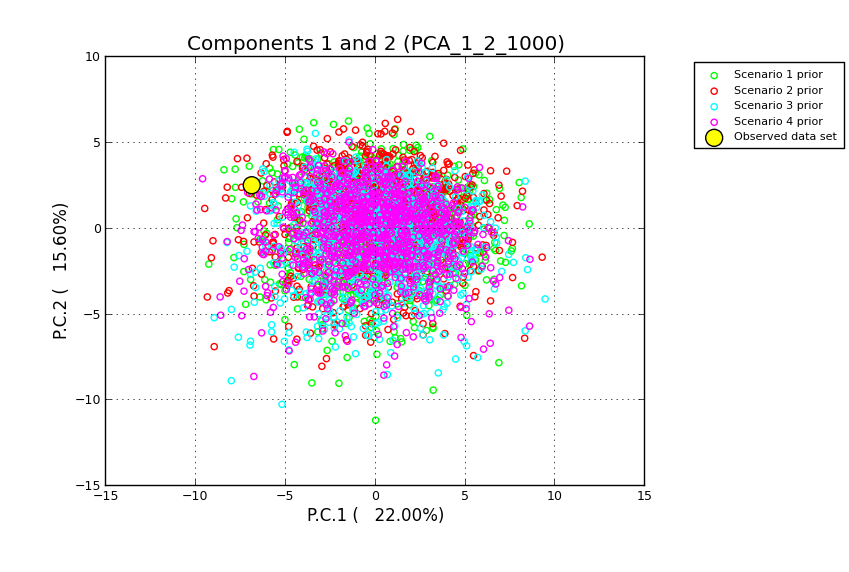


**(b)**


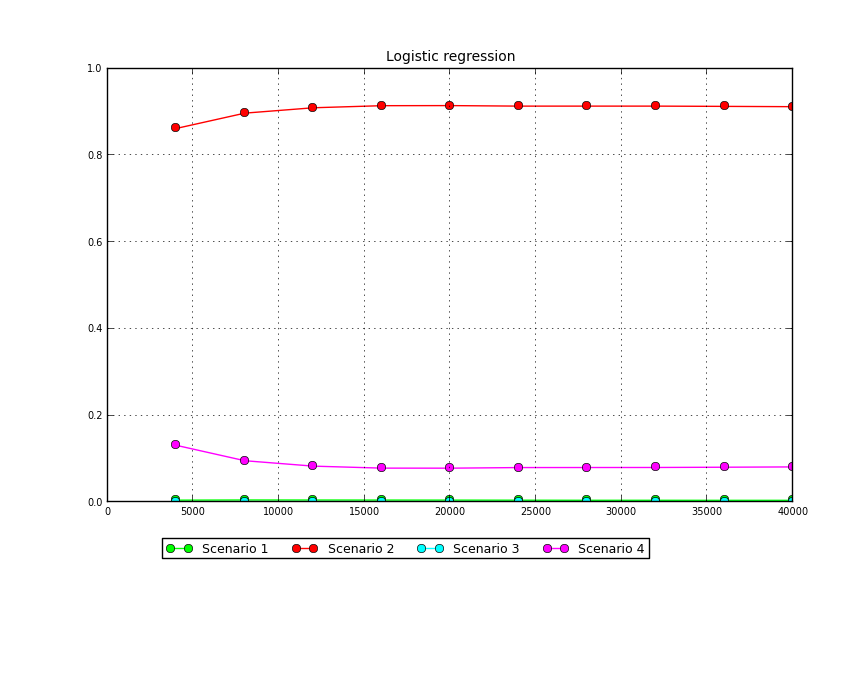


**(c)**


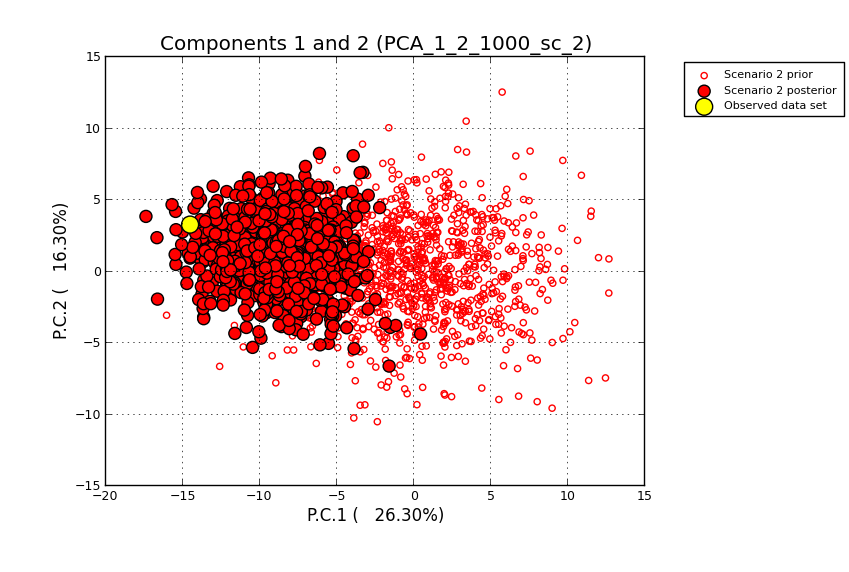


**Fig. S4** Results of the DIYABC analyses for the South East Asian populations (Indonesia).

**(a):** principal component analysis (PCA) to check that at least one combination of scenarios and priors can produce simulated data sets that are close enough to the observed data; **(b):** model comparison based on a logistic regression approach; **(c):** model checking with PCA to evaluate how well the best scenario and parameter posterior distribution combinations fit the observed data.

**(a)**


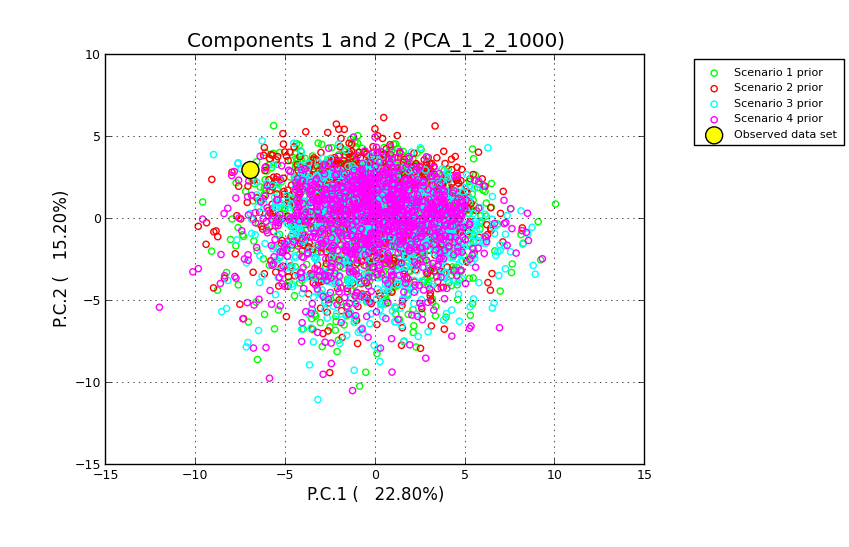


**(b)**


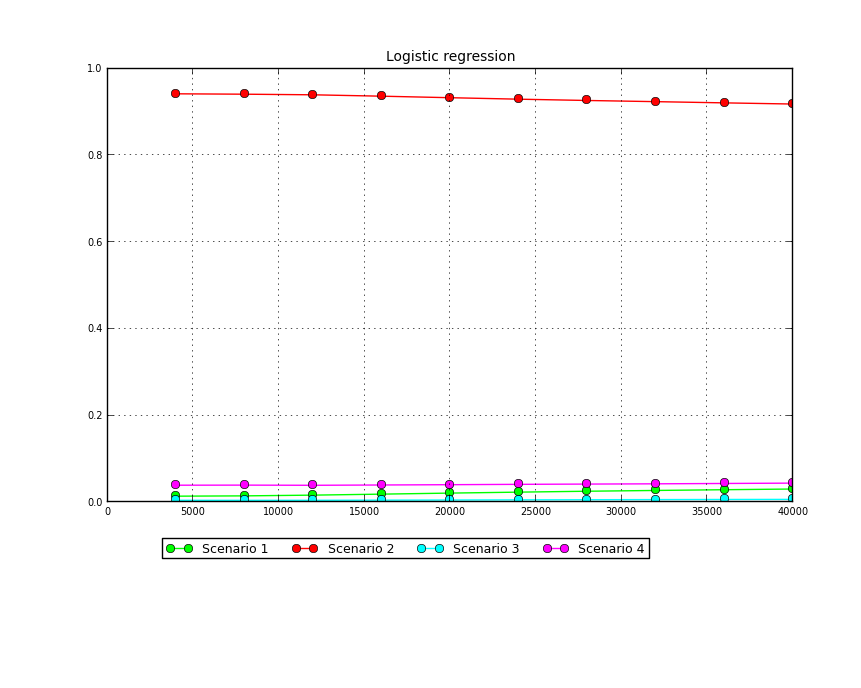


**(c)**


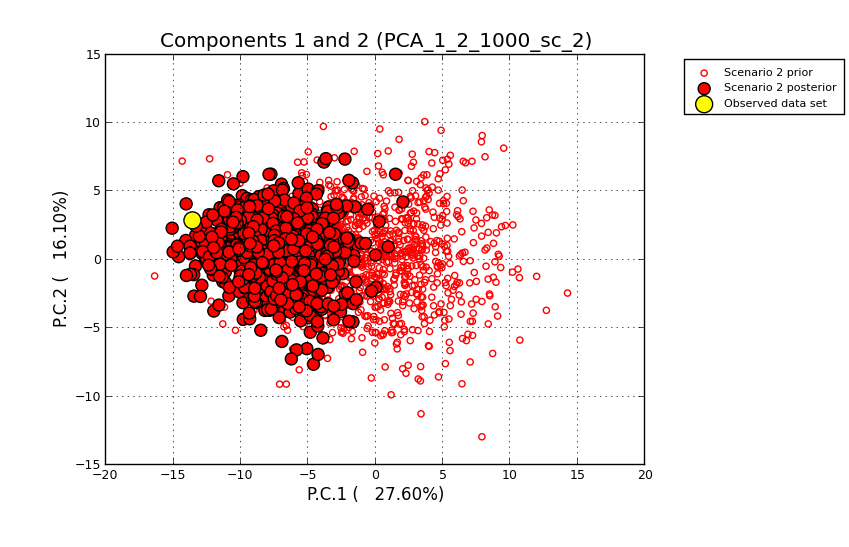


**Fig. S5** Results of the DIYABC analyses for the West Asian population (Lebanon).

**(a):** principal component analysis (PCA) to check that at least one combination of scenarios and priors can produce simulated data sets that are close enough to the observed data; **(b):** model comparison based on a logistic regression approach; **(c):** model checking with PCA to evaluate how well the best scenario and parameter posterior distribution combinations fit the observed data.

**(a)**


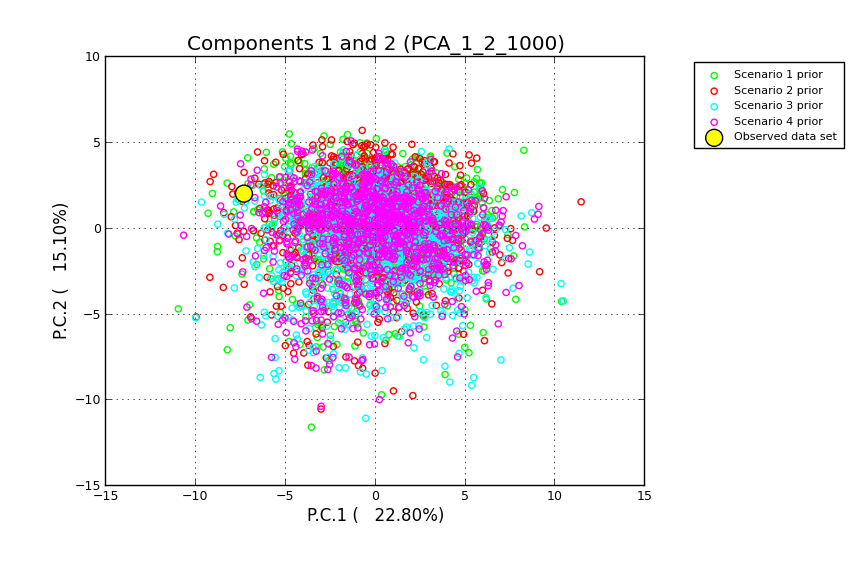


**(b)**


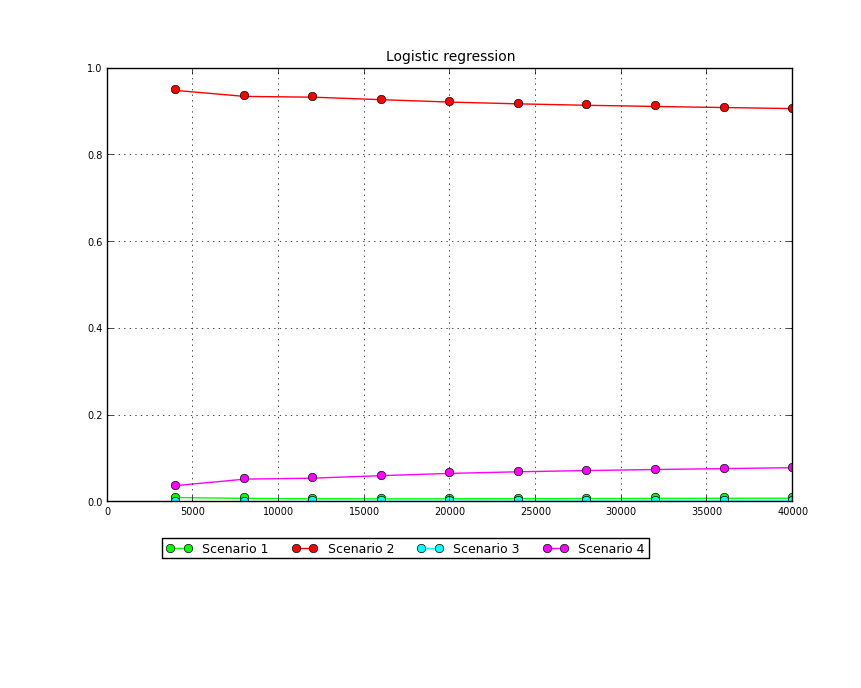


**(c)**


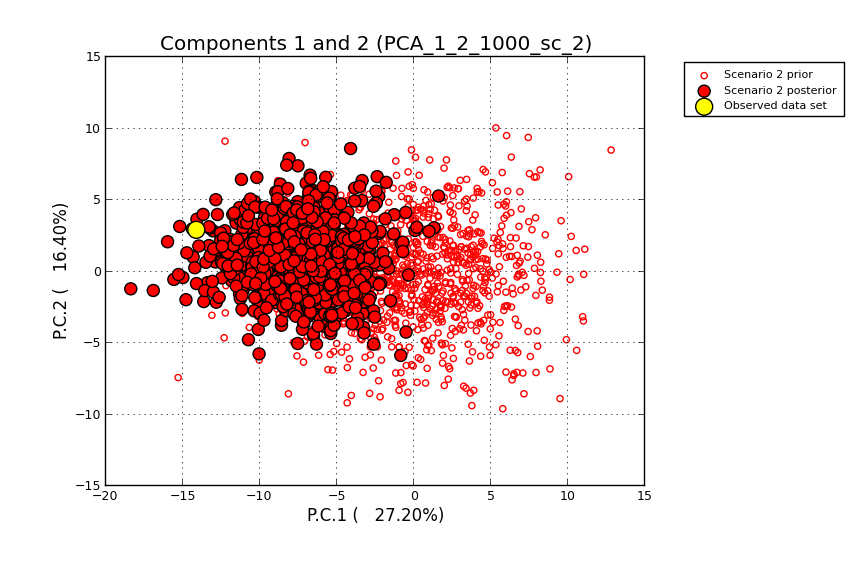


**Fig. S6** Results of the DIYABC analyses for the East Asian population (Japan).

**(a):** principal component analysis (PCA) to check that at least one combination of scenarios and priors can produce simulated data sets that are close enough to the observed data; **(b):** model comparison based on a logistic regression approach; **(c):** model checking with PCA to evaluate how well the best scenario and parameter posterior distribution combinations fit the observed data.

**(a)**


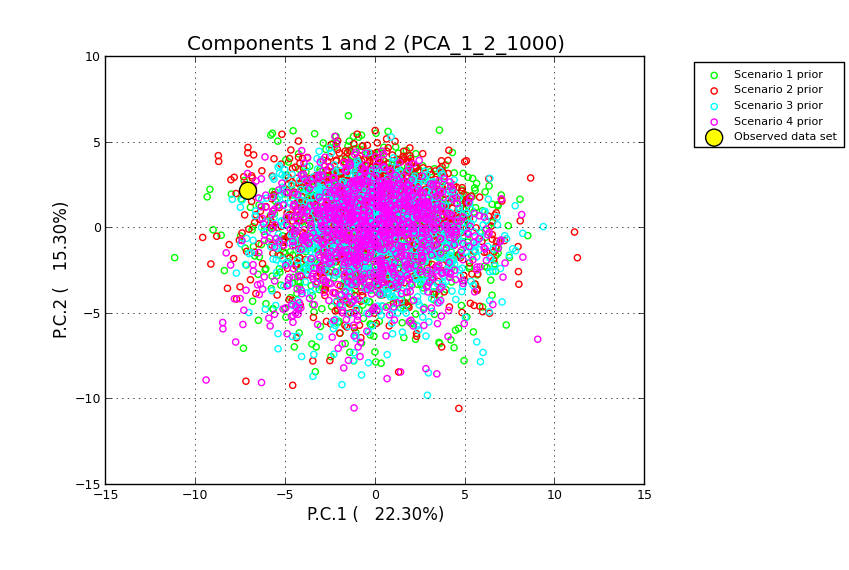


**(b)**

**
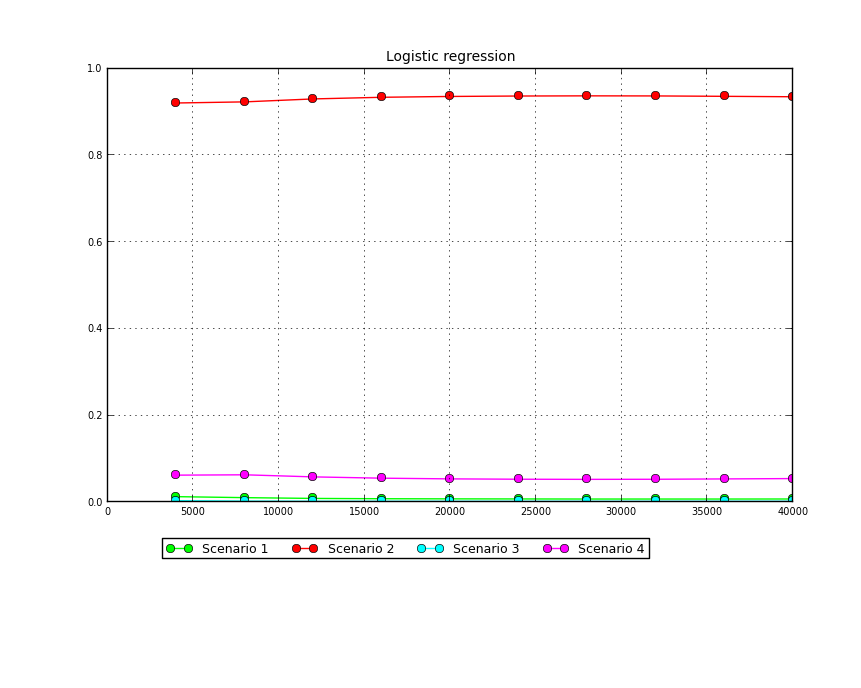
**

**(c)**

**
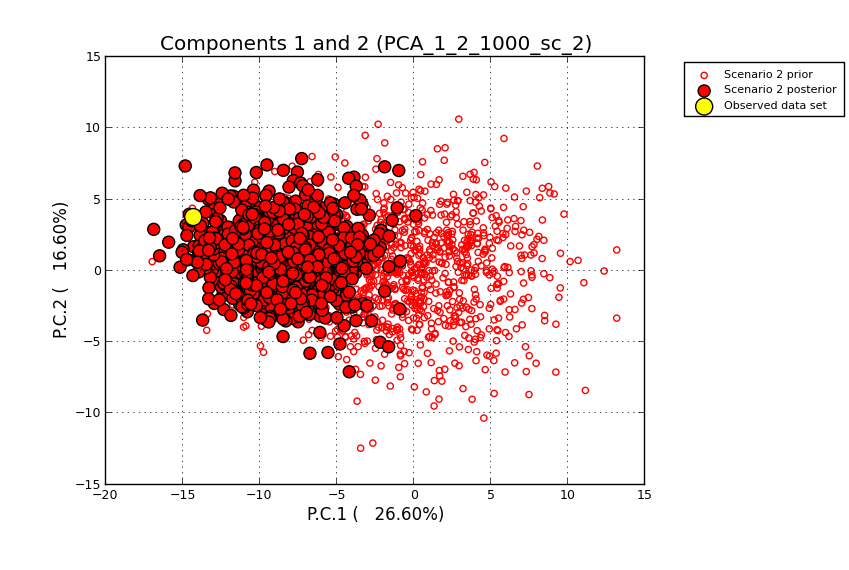
**

**Fig. S7** Results of the DIYABC analyses for the Australian populations (Australia).

**(a):** principal component analysis (PCA) to check that at least one combination of scenarios and priors can produce simulated data sets that are close enough to the observed data; **(b):** model comparison based on a logistic regression approach; **(c):** model checking with PCA to evaluate how well the best scenario and parameter posterior distribution combinations fit the observed data.
